# How does CSR of food company affect customer loyalty in the context of COVID-19: a moderated mediation model

**DOI:** 10.1186/s40991-021-00068-4

**Published:** 2022-01-17

**Authors:** N. Zhang

**Affiliations:** Cardiff Business School, Cardiff University, Aberconway Building, Colum Drive, CF10 3EU Cardiff, United Kingdom

**Keywords:** CSR, Customer loyalty, COVID-19, Moderating effects

## Abstract

Because of COVID-19 in the world, enterprises and consumers pay more and more attention to environmental protection, food safety and health issues. The purpose of this paper is to take China's food company as an example to study the impact of CSR on customer loyalty, mediating effects of company image and customer satisfaction, and moderating effects of COVID-19. The result shows that during COVID-19, company image and customer satisfaction have significant mediating effects, and COVID-19 positively moderate the impact of CSR on customer satisfaction.

## Introduction

In China, with the progress of science & technology and the improvement of people's living standards, people's views on food consumption have changed. Consumers pay more and more attention to corporate social responsibility (CSR) in environment and product safety (Ge 2018). In 2008, for example, China's Sanlu Group received repeated complaints from consumers that its milk powder contained substances that were harmful to human health. Later, Government Quality Inspection Department found that the company's milk powder contained "melamine" which was harmful to the child. The 'melamine' incident which fermented in September 2008 was a food safety scandal that shocked both at home and abroad. In the same way, public health events such as SARS in 2003 and the current COVID-19 epidemic in the world have made enterprises and consumers pay more and more attention to environmental protection, food safety and health. Therefore, studying the impact of social responsibility in the food industry on customer loyalty, and whether public health events play a moderating role in this impact, it is very necessary to understand the characteristics of consumer behaviors and CSR.

The purpose of this paper is to take China's food company as an example to study the impact of CSR on customer loyalty. The research literature on the relationship between customer loyalty and CSR is rich, and most of them believe that CSR has a significant impact on customer loyalty (Osakwe and Yusuf 2020). However, although different scholars have studied different industries, there are few studies on China's food industry. Moreover, due to the global COVID-19 epidemic in 2020, this public health event has changed people's consumption patterns and views on CSR, so the innovation of this paper is to pay attention to China's food companies and the moderating role of COVID-19. The second part is a literature review and hypotheses, the third part is model and variable description, the fourth part is questionnaire design and data analysis. Finally, the conclusion is given.

## Literature Review

### CSR, Customer Loyalty, Corporate Image and Customer Satisfaction

#### CSR

Ever since Sheldon (1924) first defined CSR, many scholars have discussed it from different views. From the enterprises’ perspective, Sheldon argues that enterprises should bear all kinds of social influence from the perspective of ethical events. According to the Council of Europe (2011), CSR is a social influence, an important enterprise resource considered to affect customers’ perception of the enterprise (Yuen et al. 2016). Frederick (1983) put forward spontaneous and compulsory social responsibility. Spontaneous social responsibility tends to charity donation, while compulsory social responsibility refers to the responsibility stipulated by laws and regulations. Carroll (1991) expanded the above contents, dividing the CSR from low to high into four aspects, namely, economic responsibility, legal responsibility, ethical responsibility and charitable responsibility, and describing them in the form of CSR pyramids. By synthesizing concepts of CSR, (Yang and Guo 2014) argue that social responsibility has evolved from ethical philosophy, corporate sustainability, ethics and values to religion.

From the stakeholder’s perspective, (Brown and Dacin 1997) argue that although enterprises still have the responsibility to shareholders in the past, more attention needs to be paid to social responsibility to other stakeholders, including employees, consumers, communities, customers, governments, etc. (Sarkar and Searcy 2016) divide CSR into three stages by analysing the evolution process and synthesizing the research contents of the three stages. They believe that CSR includes economic responsibility, social responsibility, ethical responsibility, stakeholder responsibility, sustainability and so on. According to Han et al. (2020)’s analysis, CSR is a comprehensive concept, including active social responsibility activities, passive social response, and some contributions beneficial to society.

#### Customer Loyalty

Customer loyalty is a stable and lasting relationship (Singh and Sirdeshmukh 2000) between the customer and the product or service provider. For customers, customer loyalty is a persistent commitment for the customer to form a positive word of mouth after multiple purchases of the product or service (Chanu et al. 2009). Kim (2011) believes that the company's loyal customers generally have a feeling of love or attachment to the company's products. The external performance of loyalty includes the size of purchase share, repeat purchase behaviour and so on, but scholars prefer to use behaviour scale to evaluate customer loyalty, such as how environmental changes affect customer behaviour loyalty (Jarideh 2016 Aghamolaei et al. 2014).

From the perspective of corporate development, customer loyalty is an important factor affecting corporate success and profitability, so enterprises often regard customer loyalty as a critial goal of their long-term development (Srivastava and Rai 2014). In other words, customer loyalty seems to be invisible, but it has great commercial value. Especially when enterprises are faced with the external adverse environment such as buyer's market, loose customer relationship and increased competitive pressure, customer loyalty will show an important strategic position (Srivastava and Rai 2018). For an enterprise, once customer recognizes their product or service and has certain psychological dependence (Cui 2014), they will also actively recommend trusted products and do free word-of-mouth publicity for the enterprise (Liu 2015). Therefore, repeated purchase and word of mouth recommendations become an important index to measure customer loyalty (Zhang 2019).

#### Corporate Image

Corporate image is an abstract concept, which reflects the public's overall evaluation of the enterprise (Guo and Liu 2004). As the concept of corporate image is very broad, there are some differences for different scholars. From the perspective of stakeholders, the corporate image is the stakeholders' perception of corporate social concerns (Lai et al. 2010). As an intangible resource, corporate image can strengthen consumers' attitudes and behaviour (Perez and Rodriguez del Bosque 2015b). From the perspective of corporate marketing, marketing strategy is a potential resource method that includes opportunities and threats in the decision-making process, which is mainly used to achieve effective marketing goals (Pribadi and Rivai 2020). Therefore, to achieve consumer satisfaction and improve corporate image, the corporate marketing strategy will focus on all kinds of CSR-related public relations activities or emotional marketing activities. (Mukonza and Swarts 2020) studied the impact of green marketing strategy on corporate performance and corporate image and found that it can effectively improve the image of the company, and promote more consumers to buy green products (Yunus and Rahman 2014). Therefore, the company image is the index that the enterprise pays close attention to in the marketing process. It is used to describe, remember and contact the company, and it is the final result of customer experience, impression, belief, feeling and knowledge (Copley 2004).

#### Customer Satisfaction

Satisfaction is an individual's response to feeling, which is the degree to which consumers perceive the expectations of a product and service (Sangadji and dan Sopiah. 2013). When customers' expectations of products or services match reality, they will feel satisfied, otherwise, they will feel disappointed (Oktareza et al. 2020). For the perception in the process of consumption, the different consumer has different experiences, and different satisfaction rates are generated (Oliver 1997). During the marketing process, customer satisfaction is often the focus of the company strategy.

### Relations and Hypothesis

Customers, as important stakeholders of enterprises, are concerned by enterprises. Customer loyalty is one of the most basic behaviours that enterprises hope to influence customers by using CSR as a marketing tool. Although some researchers question the direct relationship between CSR actions and loyalty or consumers' future behaviour intention (Lombart and Louis 2014), most scholars argue that there is a positive correlation between CSR and loyalty (Mohr and Webb 2005). For example, (Servera-Francés and Piqueras-Tomás 2019) found that CSR has a positive impact on consumer loyalty through consumers' perceived value. Zhang (2019) found that charitable responsibility and customer responsibility have a positive impact on customer loyalty. From an industry point of view, taking retail banks as an example, Osakwe and Yusuf (2020) study the roadmap of customer loyalty, take reputation and trust as intermediary variables, and find that CSR has a positive impact on customer loyalty. Other scholars analyse the positive impact of CSR on customer loyalty from the perspective of the real estate industry (Sun 2018), hotel industry (Latif et al. 2020), bank industry (Perez and Rodriguez del Bosque 2015a) and dairy industry (Chen 2014).

Generally speaking, corporate image originates from the performance of CSR, and it is also a tool to create a good corporate image (Martinez et al. 2014). From the perspective of CSR, CSR can not only improve the positive image of the enterprise but also help the enterprise to resist the negative influence (Ge 2018). (Rowley and Berman 2000) believe that corporate investment in CSR can enhance corporate reputation and brand image. Kim et al. (2020) found that CSR (economic, ethical, legal, and philanthropic) has a positive impact on corporate image and customer citizen behaviour.

CSR not only affects the image of the company, but it will also directly affect customer satisfaction. Many scholars believe that companies that take on CSR positively contribute to customer satisfaction (Rowley and Berman 2000, Jayachandran et al. 2005, He and Li 2011, Hsu 2012). The main reason is that a good record of CSR improves customers' evaluation of the company, and customers will get higher satisfaction from responsible company products (Luo and Bhattacharya 2006).

So, the first hypothesis is,

- **H1: CSR has a positive impact on customer loyalty, corporate image and customer satisfaction.**

Corporate image shows two kinds of characteristics (Dowling 2004). One is the ability and financial performance of the company, the other is the more emotional drive, such as social responsibility and the unique personality of the organization. As a company shows a strong and convincing good image of the company through marketing, it will attract more attention and psychological needs of customers, thus achieving customer satisfaction (Kotler and Keller 2012). Moreover, satisfied customers tend to spread positive word of mouth (Anderson 1998) and purchase intention (Anserson and Mittal 2000), and further achieve customer loyalty. In addition, the company image is a mirror of the enterprise, loyal customers are willing to buy the same product again and again, showing high customer loyalty (Yazid et al. 2020). So, the second and third hypotheses are,

- **H2: Company image has a positive impact on customer satisfaction and customer loyalty.**
- **H3: Customer satisfaction has a positive impact on customer loyalty.**

### Further analysis in Chinese food market

The food industry is special, and the protection of food safety is the basic social and legal responsibility that enterprises should bear first. According to the China Food Industry Social Responsibility Research and Analysis Report (2020), with the increasing improvement of China's food safety laws and regulations and the strengthening of food safety supervision, many food enterprises actively issue annual social responsibility reports, disclose CSR activities. However, the main problem of social responsibility in China's food industry is that it is difficult to manage the supply chain. Mainly because the food industry involves many industries and links, such as agriculture, animal husbandry, fishery, food processing, packaging and transportation, it is difficult to comprehensively manage the food safety problems arising from each link. Once a food company violates social responsibility, it will affect the corporate image and customer loyalty of the whole supply chain (Liu et al. 2015a, b).

There are three main aspects of CSR research on Chinese food enterprises. One is the research on the relationship between CSR and corporate performance. It is considered that enterprises that actively assume social responsibility will improve financial performance (Chen and Liu 2020, Niu and Zhou 2021). The second is about the impact of CSR information disclosure on corporate social reputation, that the more adequate CSR information disclosure, the higher corporate image and customer satisfaction will be (Zhong 2020, Gong 2020). The third is the case study on the crisis of social responsibility of food enterprises, including the scandal of Sanlu, the financial fraud of rising Coffee, etc. (Wang 2017, Dai, 2019). The case study found that there is an obvious positive relationship between CSR and corporate customer loyalty (Hu et al. 2012). So, the above hypothesizes are applied to the food industry in China.

### The moderating role of COVID-19

Since the end of 2019, the COVID-19 epidemic has had a devastating impact on the world economy and has also triggered some scholars' discussions on the relation between the COVID-19 epidemic and CSR. Qiu et al. (2021) (Ding et al. 2020) studied the impact of CSR on corporate performance under the COVID-19 epidemic and found that CSR activities help attract the attention of stakeholders such as customers. In order to improve the return of corporate stocks or slow down the decline of corporate stocks, (Manuel and Herron 2020)’s research found that firms were involved in a wide range of charitable CSR actions during the pandemic, possibly out of utilitarian and moral factors to meet the needs of internal and external stakeholders. (Bae et al. 2021) believe that if the CSR rating is out of line with actual action, companies that are open to social responsibility are not immune to the adverse effects of the crisis under the COVID-19 epidemic. By the study of hotel CSR marketing, Huang and Liu (2020) argue that the donation appeal under the COVID-19 epidemic environment has a positive impact on the brand loyalty of enterprises. (Cui and Guo 2020) argue that consumers' perception of dairy CSR during the period of COVID-19 will produce internal responses such as identity emotion, consumer trust, corporate reputation, product quality perception, and external responses such as purchase intention. Based on this literature, it can be found that public health events such as COVID-19 will strengthen customers' understanding of CSR and brand loyalty. In summary, COVID-19 strengthen all hypotheses above.

- **H41: COVID-19 positively moderate the impact of CSR on corporate image.**
- **H42: COVID-19 positively moderate the impact of CSR on customer satisfaction.**
- **H43: COVID-19 positively moderate the impact of CSR on customer loyalty.**

## Model, questionnaire and variables

### Model

**Fig. 1:**
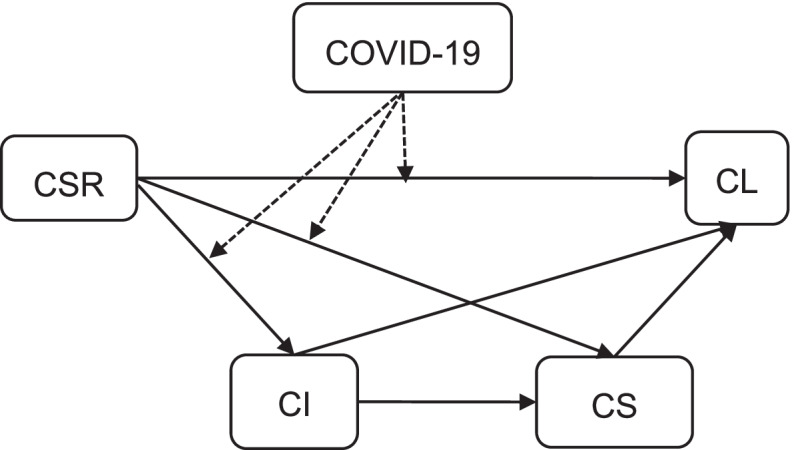
Research Model

According to the above hypothesis, Fig. 1 shows the model. CSR is an independent variable, customer loyalty (CL) is a dependent variable. Corporate Image (CI) and customer satisfaction (CS) play a mediating role, while COVID-19 play a moderating role.

### Questionnaire and variables

The survey object of the questionnaire is the largest meat company in China. A self-administered questionnaire was used in this research. The questionnaire involved five sorts of scales: CSR, company image, customer satisfaction, customer loyalty and public health events. When designing the scale of CSR, scholars mostly pay attention to the economic responsibilities, legal responsibilities, ethical responsibilities and discretionary responsibilities (Carroll 1979). Discretionary responsibilities mainly refer to the voluntary charitable responsibility of the enterprise. This study combined with the research of Carroll (1979) and Sandhu & Kapoor (2010) to design the scale. According to the research of Ishaq (2012) and Yeo et al. (2011), the questionnaire of company image is set up, the questionnaire of customer satisfaction is set up according to the study of (Kaur and Soch 2012), and the questionnaire of customer loyalty is set up according to the study of Ishaq (2012). According to the study of (Manuel and Herron 2020), Huang and Liu (2020) and (Cui and Guo 2020), the scale of COVID-19 is set up.

The questionnaire mainly aims at five kinds of questions. The first question is CSR, including economic responsibility (CSRE), legal responsibility (CSRL), ethical responsibility (CSRM), charitable responsibility (CSRC) and environmental responsibility (CSRG). The second is the corporate image (CI). The third is customer satisfaction (CS). The fourth is customer loyalty (CL). The fifth category is COVID-19. At the end of the questionnaire, there are demographic questions, including Gender, Age, an education level (Edu), profession (Prof) and Income (Table 1).

**Table 1:** Measurement Items

Primary Variable	Secondary Variable	Measurement Items of questionnaire
CSR	CSRE	CSRE1: The company has a strong competitive advantage
		CSRE2: The company is a profitable enterprise
		CSRE3: The company has strong economic strength
		CSRE4: The company is committed to maximizing profits
	CSRL	CSRL1: The company abides by local and national laws
		CSRL2: The company operates in accordance with government regulations
		CSRL3: The safety of the company's products is in line with national laws and regulations
		CSRL4: The company fulfils its legal obligations imposed by the state or community
	CSRM	CSRM1: The company strives to be a good citizen
		CSRM2: The company is ethical in its treatment of employees, consumers, communities and other stakeholders
		CSRM3: The company's products are in line with ethical norms in terms of safety and green
		CSRM4: The company can operate in line with social and ethical expectations
	CSRC	CSRC1: The company actively participates in charitable activities such as relief, poverty alleviation and donation
		CSRC2: The company is committed to supporting the improvement of the quality of life in the community
		CSRC3: The company supports and promotes philanthropy
	CSRG	CSRG1: The company actively carries out green certification of its products
		CSRG2: The company actively participates in community environmental protection
		CSRG3: The company actively participates in community public health services
		CSRG4: The company pays attention to energy saving and emission reduction in the production process
CI		CI1: The company has a good social reputation
		CI2: The company is an example of food safety
		CI3: The company has a high degree of transparency
		CI4: The company has a good reputation among consumers
CS		CS1: The company's CSR policy caters to my expectations
		CS2: I am very satisfied with the CSR performance of the company
		CS3: I am satisfied with the products and services of the company
CL		CL1: I am willing to recommend this company to the people around me
		CL2: I would like to patronize this company
		CL3: I would like to recommend the products of this company to my friends or colleagues
COVID-19		COVID1: During COVID-19, the enterprise should abide by the epidemic prevention policy of the community
		COVID2: During COVID-19, the enterprise should bear the corresponding charitable responsibility
		COVID3: During COVID-19, the enterprise should bear the ethical responsibility to the stakeholders
		COVID4: During COVID-19, the enterprise should actively participate in the environmental health service activities of the community
		COVID5: If the company undertakes more social responsibility during COVID-19 epidemic, my loyalty to the company will be enhanced
		COVID6: During COVID-19, the enterprise should abide by the epidemic prevention policy of the community

The calculation relationship between variables is shown in Table 2:

**Table 2:** Calculation relationship between variables

Variables	Calculation relationship
CSR	CSR = (CSRE + CSRL + CSRM + CSRC + CSRG)/5
	CSRE = (CSRE1 + CSRE2 + CSRE3 + CSRE4)/4
	CSRL = (CSRL1 + CSRL2 + CSRL3 + CSRL4)/4
	CSRM = (CSRM1 + CSRM2 + CSRM3 + CSRM4)/4
	CSRC = (CSRC1 + CSRC2 + CSRC3)/3
	CSRG = (CSRG1 + CSRG2 + CSRG3 + CSRG4)/4
CI	CI = (CI1 + CI2 + CI3 + CI4) /4
CS	CS = (CS1 + CS2 + CS3)/3
CL	CL = (CL1 + CL2 + CL3)/3
COVID-19	COVID-19 = (COVID1 + COVID2 + COVID3 + COVID4 + COVID5 + COVID6)/6

## Data Analysis

### Demographic analysis

This survey sends out 265 electronic questionnaires, including 247 valid questionnaires and 18 invalid questionnaires. The demographic information includes gender, age and income. According to the one-way ANOVA of CSR by gender, it is found that the corresponding P-value is higher than the significant level of 0.05. That is to say, CSR is not affected by gender. But in the one-way ANOVA of CSR by age and income, it is found that the corresponding P values is less than 0.05 and 0.1 respectively, CSR is affected by them (Table 3).

**Table 3:** One-way ANOVA CSR by gender, age and income

Variables	Sum of squares	df	Mean square	F	P-value
Gender					
Between groups	0.044	1	0.044	0.240	0.624
Within the group	44.613	245	0.182		
Sum	44.657	246			
Age					
Between groups	2.388	5	0.478	2.723	0.021
Within the group	42.269	241	0.175		
Sum	44.657	246			
Income					
Between groups	1.958	5	0.392	2.211	0.054
Within the group	42.698	241	0.177		
Sum	44.657	246			

**Fig. 2:**
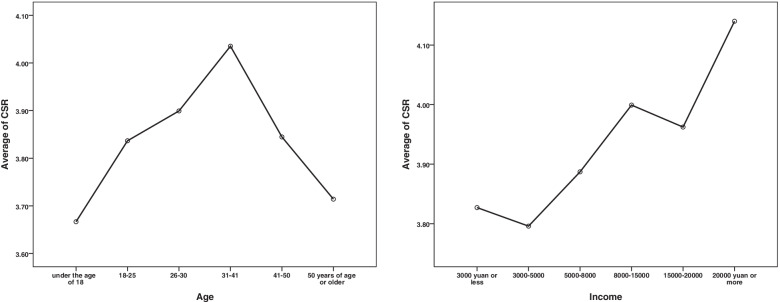
Mean Graph by Age and Income Group

According to the mean map of age and income group, it can be found that the respondents aged between 31 and 41 have the highest CSR score, while the younger and older respondents score lower and pay less attention to CSR. And with the increase of income, the respondents have the highest score on CSR, that is, the attention to CSR increases with the increase of income (Fig. 2).

### Reliability and Validity tests

The reliability test mainly focuses on the Cronbach coefficient based on the standardization term, which ranges from 0 to 1, and the closer it is to 1, the higher the reliability. As can be seen from Table 4 below, the reliability analysis of all the dimensions of the five measures of CSR, corporate image, customer satisfaction, customer loyalty and COVID-19 show that the value of the index Cronbach's Alpha is greater than 0.5, indicating that the questionnaire is reliable (Streiner 2003; Ekolu and Quainoo 2019).

**Table 4:** Reliability Statistics

	Cronbach's Alpha	Cronbach's Alpha Based on Standardized Items	N of Items
**CSR**			
Economic Responsibility	0.574	0.588	4
Law Responsibility	0.711	0.712	4
Moral Responsibility	0.698	0.697	4
Charity Responsibility	0.711	0.712	3
Environment Responsibility	0.711	0.712	3
**Corporate Image**	0.774	0.777	4
**Customer Satisfaction**	0.745	0.744	3
**Customer Loyalty**	0.769	0.773	3
**COVID-19**	0.765	0.764	6

As for validity analysis, this paper mainly focuses on KMO and Bartlett's Test. As can be seen from Table 5 below, the KMO coefficient is 0.908, which is very close to the significance level of 0.000, less than 0.05 for the test of Sphericity of the Personality Bartlettings. So, the questionnaire passed the validity test (Shrestha 2021, Tabachnick and Fidell 2013).

**Table 5:** KMO and Bartlett's Test

Kaiser–Meyer–Olkin Measure of Sampling Adequacy	0.908
Bartlett's Test of Sphericity	
Approx. Chi-Square	3650.133
df	595
Sig	0.000

### Mediating and moderating effects

The mediator variable explains the relationship between an independent variable and a dependent variable, exhibiting indirect causation, connection, or relation. The moderator variable alters the relation between two variables, and thus modifies the form or strength of the relationship between an independent and dependent variable (Gómez et al. 2020).

#### Model description

In the analysis of mediating effect, moderating effect and conditional process in Hayes (2017), 92 models are given. In this paper, the model 85th in the process is adopted according to the problem studied, and the process program in SPSS software is used for analysis. The corresponding relationship between model variables and research variables is shown in Fig. 1 above. In addition, age, education, and profession are taken as control variables.

#### Result analysis

According to the above model, the variables are standardized and analyzed by process program in SPSS. There are three equations (Table 6) which show the causal relationships among variables and the moderating effect of COVID-19. The effects of CSR on CI, CSR and CI on CS, CSR, CI and CS on CL, are all significant. The moderating effect of COVID-19 is significant in Eq. 2, not significant in Eq. 1 and Eq. 3.

**Table 6:** Moderated mediation model test

	Equation 1 (Dependent variable: CI)			Equation 2 (Dependent variable: CS)			Equation 3 (Dependent variable: CL)		
Variable	β	se	t	β	se	t	β	se	t
constant	0.199	0.345	0.576	0.075	0.313	0.238	0.160	0.341	0.469
CSR	0.637^***^	0.051	12.409	0.441^***^	0.060	7.400	0.219^***^	0.072	3.046
CI				0.380^***^	0.059	6.502	0.332^***^	0.069	4.817
CS							0.270^***^	0.070	3.841
COVID-19	0.134^**^	0.054	2.463	0.064	0.050	1.278	-0.029	0.054	-0.536
CSR*COVID-19	0.000	0.043	-0.002	0.078^**^	0.039	2.002	0.004	0.043	0.097
Age	-0.041	0.045	-0.915	-0.018	0.040	-0.448	0.043	0.044	0.982
Edu	-0.052	0.106	-0.496	0.010	0.096	0.100	-0.089	0.104	-0.856
Prof	0.016	0.013	1.174	-0.012	0.012	-0.940	-0.007	0.013	-0.502
R^2^	0.512			0.601					
F	41.949^***^			51.446^***^					

Note: * *p* < 0.1; ** *p* < 0.05; *** *p* < 0.01

**Fig. 3:**
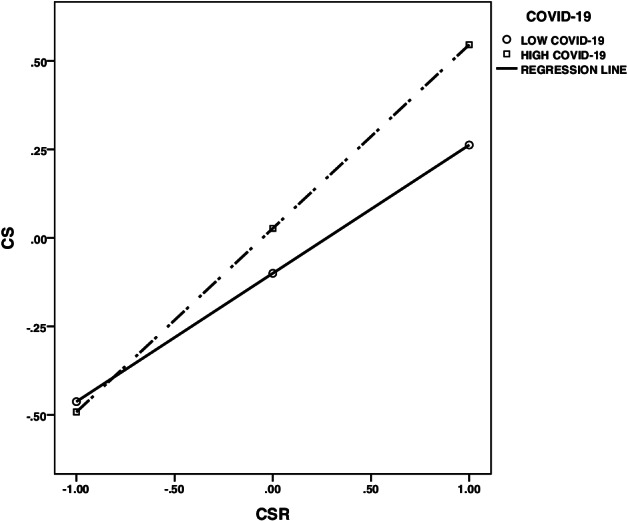
The moderating effects of COVID-19 between CSR and CS

To more clearly explain the nature of the interaction effects between CSR and COVID-19, COVID-19 was grouped by the value of the mean plus or minus one standard deviation, a simple slope test was performed, and a simple effect analysis diagram was plotted (Fig. 3).The results shows that CSR has a positive predictive effect on CS for low group (β = 0.36, t = 5.13, p < 0.001) and high group (β = 0.52, t = 7.23, *p* < 0.001), and the positive predictive effect of CSR on CS are strengthened in high group.

Therefore, according to the above analysis of the mediating effect of CI and CS and the moderating effect of COVID-19, the test results of the five basic hypotheses are as follows (Table 7):

**Table 7:** Results of test

Hypothesis	Support/ no support
H1	Support
H2	Support
H3	Support
H41	No support
H42	Support
H43	No support

## Discussion

According to geographic analysis (Table 3), age and income rather than gender affected perceptions of CSR. This is mainly because working middle-aged people are more likely to feel the importance of CSR. In addition, since the quality of consumers is positively correlated with their income, with the increase of income and consumers’ quality, consumers pay more attention to CSR.

According to mediating analysis (Table 6), H1, H2 and H3 are tested. These conclusions are consistent with most scholars. For mediating analysis, H42 is tested, but H41 and H43 are not tested. This conclusion is new because COVID-19 is a worldwide public health event that reinforces the positive impact of CSR on customer satisfaction. However, its influence on customer loyalty and corporate image has not been strengthened. The main reason is that during COVID-19, consumers, as the victim of Coronavirus, were in a nervous state for a long time. Customers believed that CSR was what enterprises should do, which could only make consumers more satisfied but could not make consumers more loyal to the company.

## Limitations and further research

The limitations of the paper are mainly shown in the following aspects. First of all, the selection of variable dimensions is not comprehensive. For example, in the dimension selection analysis of CSR, only six dimensions are selected, while CSR contains many dimensions of responsibility in reality. Secondly, this paper refers to the 82nd model of Hayes (2017) and makes an empirical analysis, and doesn’t expand the model meaningfully from different angles. Finally, in issuing a questionnaire, it is unable to control the identity of the respondents, they will face the respondents' lack of understanding of the concept of CSR in the process of collection, which has an impact on the quality of the data to some extent.

It is hoped that there will be some development and breakthroughs in the following aspects in the future. Firstly, in the future, according to the characteristics of different industries, this paper can design targeted models to give guidance to various industries in the implementation of CSR other than the food industry. Secondly, we can choose a specific consumer group to carry out analysis in the future and to further enrich the study of CSR. Finally, other stakeholders can be studied. CSR affects not only consumers but also other stakeholders. Therefore, future research can be carried out from the perspective of different stakeholders.

## Conclusions

According to the above empirical analysis, three conclusions can be drawn. (1) CSR is not affected by gender but is affected by age and income level. Middle-aged people aged between 31 and 41 pay the highest attention to CSR. With the increase of income, consumers' attention to CSR is increasing. (2) CSR has a positive impact on customer loyalty, corporate image and customer satisfaction. Company image and customer satisfaction have significant mediating effects. (3) COVID-19 positively moderate the impact of CSR on customer satisfaction, but no positively moderation for the impact of CSR on customer loyalty and corporate image.

## Data Availability

Data were collected from questionnaires. The data format is SPSS file. See the link: https://pan.baidu.com/s/1XMo3Ndd4gmd9ToElOfFhew. Password: 9mey.

## Abbreviations

CSR: Corporate social responsibility
COVID-19: Corona Virus Disease 2019
SARS: Severe Acute Respiratory Syndrome
CL: Customer loyalty
CI: Corporate image
CS: Customer satisfaction
ANOVA: Analysis of variance
KMO: Kaiser–Meyer–Olkin

## References

[CR1] Aghamolaei T, Eftekhaari TE, Rafati S, Kahnouji K, Ahangari S, Shahrzad ME, Kahnouji A, Hoseini SH (2014). Service quality assessment of a referral hospital in Southern Iran with SERVQUAL technique: Patients’ perspective. BMC Health Services Research.

[CR2] Anderson EW (1998). Customer satisfaction and word of mouth. Journal of Service Research..

[CR3] Anserson EW, Mittal V (2000). Strengthening the satisfaction-profit chain. Journal of Service Research.

[CR4] Bae, K. .H. ., El Ghoul, S., & Gong, Z. (2021). Does CSR matter in times of crisis? Evidence from the COVID-19 pandemic. *Journal of Corporate Finance,**67*, 101876.

[CR5] Brown TJ, Dacin PA (1997). The company and the product: Corporate associations and consumer product responses. Journal of Marketing.

[CR6] Carroll AB (1979). A Three-Dimensional Conceptual Model of Corporate Performance. Academy of Management Review.

[CR7] Carroll AB (1991). The pyramid of corporate social responsibility: Toward the moral management of organizational stakeholders. Business Horizons.

[CR8] Carroll AB, Shabana KM (2010). The business case for corporate social responsibility: A review of concepts, research and practice. International Journal of Management Reviews.

[CR9] Chanu HH, Wang YH, Yang WY (2009). The Impact of E-Service Ouality, Customer Satisfaction and Loyalty on E-Marketing; Moderating Effect of Perceived Value. Total Quality Management&business Excellence.

[CR10] Chen XF (2014). Empirical Analysis of the relationship between corporate social responsibility and Customer Loyalty-Based on the perspective of dairy products consumers. Scientific Research Management.

[CR11] Chen L, Liu J (2020). Research on the relationship between Social responsibility and Financial performance of Food Manufacturing Enterprises in China. Productivity Research.

[CR12] Copley, P. (2004). *Marketing communications management: Concepts and theories cases and practice*. Elsevier.

[CR13] L N Cui 2014 Z Bank Retail customer loyalty Management Research. Master's thesis of Heilongjiang University in China

[CR14] Cui LH, Guo R (2020). Consumer perception and response to dairy corporate social responsibility in the new crown pneumonia epidemic. China Dairy Industry.

[CR15] Dai ZL (2019). Research on Food Corporate Social responsibility- take Haidilao as an example. Modern Commerce and Trade Industry.

[CR16] W Ding R Levine C Lin W Xie (2020) Corporate Immunity to the COVID-19 Pandemic NBER Working Paper 27055. http://www.nber.org/papers/w27055. Accessed 1 Feb 2021. 10.1016/j.jfineco.2021.03.005 PMC8457922 34580557

[CR17] Dowling GR (2004). Journalists’ evaluation of corporate reputations. Corporate Reputation Review.

[CR18] Ekolu SO, Quainoo H (2019). Reliability of assessments in engineering education using Cronbach’s alpha, KR and split-half methods. Global Journal of Engineering Education.

[CR19] Frederick, (1983). Corporate social responsibility in the Reagan era and beyond. California Management Review..

[CR20] Ge Y.D. (2018). A study on the impact of corporate social responsibility on financial performance-with food and beverage listed enterprises as the research object. Master thesis, University of Electronic Science and Technology.

[CR21] Gómez LE, Schalock RL, Verdugo MA (2020). The role of moderators and mediators in implementing and evaluating intellectual and developmental disabilities-related policies and practices. Journal of Developmental and Physical Disabilities.

[CR22] Gong X M (2020). Research on the current situation of social responsibility disclosure of listed companies in food industry. Marketing.

[CR23] Guo XY, Liu XT (2004). Research on corporate image evaluation methods. Business Research.

[CR24] Han H, Yu J, Lee K, Baek H (2020). Impact of corporate social responsibility on customer responses and brand choices. Journal of Travel & Tourism Marketing.

[CR25] Hayes, A. F. (2017). Introduction to mediation, moderation, and conditional process analysis: A regression-based approach. Guilford publications.

[CR26] He H, Li Y (2011). CSR and service brand: The mediating effect of brand identification and the moderating effect of service quality. Journal of Business Ethics.

[CR27] Hsu KT (2012). The advertising effects of corporate social responsibility on corporate reputation and brand equity: Evidence from the life insurance industry in Taiwan. Journal of Business Ethics.

[CR28] Hu X.T., Jin W.H., Tian H.(2012) . Research on the relationship between Corporate Social responsibility and customer loyalty-- A case study of food enterprises. Wind of Science and Technology, 21: 257

[CR29] Huang H., Liu S. Q. (2020). Donate to help combat COVID-19! How typeface affects the effectiveness of CSR marketing? International Journal of Contemporary Hospitality Management, ahead-of-print(ahead-of-print)

[CR30] Ishaq I (2012). Perceived value, service quality, corporate image and customer loyalty: Empirical assessment from Pakistan. Serbian Journal of Management.

[CR31] Jarideh N (2016). The investigation of effect of customer orientation and staff service-oriented on quality of service, customer satisfaction and loyalty in hyper star stores. International Journal of Science and Research.

[CR32] Jayachandran S, Sharma S, Kaufman P, Raman P (2005). The role of relational information processes and technology use in customer relationship management. Journal of Marketing.

[CR33] Kaur H, Soch H (2012). Validating antecedents of customer loyalty for Indian cell phone users. Journal for Decision Makers.

[CR34] Kim HJ (2011). Service orientation, service quality, customer satisfaction, and customer loyalty: Testing a structural model. Journal of Hospitality Marketing & Management.

[CR35] M.J Kim X.M Yin G.M Lee 2020 The effect of CSR on corporate image, customer citizenship behaviors, and customers’ long-term relationship orientation International Journal of Hospitality Management 88 102520

[CR36] P. Kotler, K. L. Keller. 2012.Marketing Management(14e). Prentice-Hall, 163-165.

[CR37] Lai CS, Chiu CJ, Yang CF, Pai DC (2010). The effects of corporate social responsibility on brand performance: The mediating effect of industrial brand equity and corporate reputation. Journal of Business Ethics.

[CR38] Latif KF, Pérez A, Sahibzada UF (2020). Corporate social responsibility (CSR) and customer loyalty in the hotel industry: A cross-country study. International Journal of Hospitality Management.

[CR39] Liu XF, Pan LL, Sun SZ (2015). An empirical study on the relationship between customer satisfaction and loyalty in commercial banks. Price Theory and Practice.

[CR40] Liu YQ, Jiao CH, Hao XY (2015). Research on corporate social responsibility evaluation system of dairy supply chain. China Dairy Industry.

[CR41] Lombart C, Louis D (2014). A study of impact of corporate social responsibility and price image on retailer personality and consumers’ reaction-satisfaction, trust and loyalty to the retailer. Journal of Retailing and Consumer Services.

[CR42] Luo X, Bhattacharya CB (2006). Corporate social responsibility, customer satisfaction, and market value. Journal of Marketing.

[CR43] Manuel T, Herron TL (2020). An ethical perspective of business CSR and the COVID-19 pandemic. Society and Business Review.

[CR44] Martinez P, Perez A, Rodriguez-del-Bosque I (2014). CSR influence on hotel brand image and loyalty. Academia Revista Latinoamerica De Administration..

[CR45] Mohr LA, Webb DJ (2005). The effects of corporate social responsibility and price on consumer responses. The Journal of Consumer Affairs.

[CR46] Mukonza C, Swarts I (2020). The influence of green marketing strategies on business performance and corporate image in the retail sector. Business Strategy and the Environment.

[CR47] Niu JQ, Zhou CY (2021). Research on Financial Evaluation of Social responsibility of listed companies in Food Manufacturing Industry. Modern Commerce and Trade Industry.

[CR48] Oktareza MET, Halin H, Handayani S (2020). The Effect of Service on Customer Satisfaction at PT Pandu Siwi Sentosa. International Journal of Community Service & Engagement.

[CR49] Oliver, R. L. (1997). *Satisfaction: A behavioral perspective on the consumer*. McGraw Hill.

[CR50] Osakwe, C. N., Yusuf, T. O. (2020). CSR: a roadmap towards customer loyalty. Total Quality Management & Business Excellence, 1-17

[CR51] Perez A, Rodriguez del Bosque I (2015). Corporate social responsibility and customer loyalty: Exploring the role of identification, satisfaction and type of company. Journal of Services Marketing.

[CR52] Perez A, Rodriguez del Bosque I (2015). How customers construct corporate social responsibility images: Testing the moderating role of demographic characteristics. Business Research Quarterly.

[CR53] Pribadi, R. . C., & Rivai, A. (2020). The effect of emotional marketing and marketing strategy on purchase decisions through consumer satisfaction as a mediation variable in PT. Nureka Bintang Abadi. *Global Journal of Engineering and Technology Advances,**5*(3), 123–128.

[CR54] Qiu, S. .Z., Jiang, J. .N., Liu, X. .M., Chen, M. .H., & Yuan, X. .N. (2021). Can corporate social responsibility protect firm value during the COVID-19 pandemic? *International Journal of Hospitality Management,**93*, 102759. http://www.sciencedirect.com/science/article/pii/S027843192030311X%3e. Accessed 25 Jan 2021. 10.1016/j.ijhm.2020.102759 PMC9998173 36919172

[CR55] Rai AK, Srivastava M (2014). Customer Loyalty In The Indian Aviation Industry: An Empirical Examination. Asia Pacific Journal of Business and Management.

[CR56] Rowley T, Berman S (2000). A Brand New Brand of Corporate Social Performance. Business & Society..

[CR57] Sandhu HS, Kapoor S (2010). Corporate social responsibility initiatives: An analysis of voluntary corporate disclosure. South Asian Journal of Management.

[CR58] Sangadji, E.M., dan Sopiah. (2013). Prilaku Konsumen: Pendekatan Praktis Disertai: Himpunan Jurnal Penelitian. Yogyakarta: Penerbit Andi.

[CR59] Sarkar S, Searcy C (2016). Zeitgeist or chameleon? A quantitative analysis of CSR definitions. Journal of Cleaner Production.

[CR60] Servera-Francés D, Piqueras-Tomás L (2019). The effects of corporate social responsibility on consumer loyalty through consumer perceived value. Economic Research-Ekonomska Istraživanja.

[CR61] Sheldon, O. (1924). *The Philosophy of Management*. Sir Isaac Pitman and Sons Ltd.

[CR62] Shrestha N (2021). Factor Analysis as a Tool for Survey Analysis. American Journal of Applied Mathematics and Statistics.

[CR63] Singh J, Sirdeshmukh D (2000). Agency and trust mechanisms in consumer satisfaction and loyalty judgments. Journal of the Academy of Marketing Science.

[CR64] Srivastava M, Rai AK (2018). Mechanics of engendering customer loyalty: A conceptual framework. IIMB Management Review.

[CR65] Streiner DL (2003). Starting at the beginning: An introduction to coefficient alpha and internal consistency. J. of Personality Assess.

[CR66] Sun Y.Y. (2018). Research on the Influence of Corporate social responsibility in Real estate Development on Customer Loyalty. Master's degree thesis, Shandong Normal University in China

[CR67] Tabachnick, B.G. and Fidell, L.S.(2013). Using multivariate statistics (6th ed.), Pearson.

[CR68] The European Commission. (2011). Corporate social responsibility: A new definition, a new agenda for action. [online], Available from: http://europa.eu/rapid/press-release_MEMO-11-730_en.htm. Accessed 28 Jan 2021.

[CR69] Wang X.Y. (2017). Research on internal control based on corporate social responsibility. Master's degree thesis, Jilin University of Finance and Economics

[CR70] Yang L, Guo Z (2014). Evolution of CSR Concept in the West and China. International Review of Management and Business Research.

[CR71] Yazid AS, Mkheimer IBRAHIM, Mahmud MS (2020). The effect of corporate image on customer loyalty: The mediating effect of customer satisfaction. The Journal of Research on the Lepidoptera.

[CR72] Yeo R-K, Goh M, Tso S (2011). Corporate image and reputation of large mainland Chinese enterprise. Journal of Marketing Communications.

[CR73] Yuen KF, Thai VV, Wong YD (2016). Are customers willing to pay for corporate social responsibility? A study of individual-specific mediators. Total Quality Management & Business Excellence.

[CR74] Yunus M, Rahman M (2014). Green marketing for creating awareness for green consumerism. Global Disclosure of Economics and Business.

[CR75] Zhang J.Y. (2019). Research on the influence mechanism of CSR on customer loyalty. Master thesis, Zhejiang Normal University

[CR76] Zhong DY (2020). Correlation between social responsibility and financial performance of food production enterprises. Food Research and Development.

